# The value of TI-RADS combined with superb micro-vascular imagine in distinguishing benign and malignant thyroid nodules: A meta-analysis

**DOI:** 10.1371/journal.pone.0261521

**Published:** 2022-01-18

**Authors:** Changfu Zhu, Lin Zhong, Mingxin Lin, Congliang Tian, Cong Wang

**Affiliations:** 1 Ultrasound Department of the First Affiliated Hospital of Dalian Medical University, Dalian, China; 2 Pathology Department of the First Affiliated Hospital of Dalian Medical University, Dalian, China; 3 Pediatrics Department of the First Affiliated Hospital of Dalian Medical University, Dalian, China; Brigham and Women’s Hospital, Harvard Medical School, UNITED STATES

## Abstract

This meta-analysis aimed to evaluate the value of thyroid imaging report and data system (TI-RADS) combined with superb micro-vascular imagine technique(SMI) in distinguishing benign and malignant thyroid nodules. We searched PubMed, Web of Science, Cochrane Library, and Chinese biomedical databases from inception through February 31, 2021. Meta-analysis was conducted using STATA version 14.0 and Meta-Disc version 1.4 softwares. We calculated the summary statistics for sensitivity(Sen), specificity(Spe), and receiver operating characteristic (SROC) curve. Six studies that met all inclusion criteria were included in this meta-analysis. A total of 408 thyroid malignant nodules and 496 thyroid benign nodules were assessed. All thyroid nodules were histologically confirmed after SMI. The pooled Sen and Spe of TI-RADS were 0.80(95%CI = 0.71–0.87) and 0.82(95%CI = 0.75–0.87); The pooled Sen and Spe of TI-RADS combined with SMI were 0.88 (95%CI = 0.80–0.91) and 0.89 (95%CI = 0.85–0.92). The areas under the SROC curve of TI-RADS and TI-RADS combined with SMI were 0.8874(SE = 0.0165) and 0.9415(SE = 0.0102), between which there was significant difference(Z = 2.789; SE = 0.0194; *p* = 0.0053). Our meta-analysis indicates that TI-RADS combined with SMI may have high diagnostic accuracy, and is more effective than single TI-RADS in distinguishing benign and malignant thyroid nodules.

## Introduction

Thyroid cancer is a very common malignant disease, which accounts for about 1% of all cancer patients [1]. Solid thyroid nodule is a risk factor for thyroid cancer, so it is very important to differentiate thyroid nodules in time and effectively [2]. Ultrasonography has the advantage of high sensitivity in the diagnosis of thyroid nodule, and is is the first choice for clinical diagnosis and differentiation of thyroid cancer [3]. In 2017, the American College of Radiology (ACR) proposed the latest version of TI-RADS classification, ACR TI-RADS, based on large-scale evidence-based and clinical validation, which greatly promotes the differentiation of benign and malignant thyroid nodules [4]. However, due to the complexity and overlapping of the sonograms of thyroid nodules, it is still difficult to accurately identify some nodules with atypical ultrasound characteristics [5]. There are some differences between benign and malignant thyroid nodules in blood flow pattern and vascular morphology, which is helpful to distinguish benign and malignant thyroid nodules [6]. Color Doppler flow imaging (CDFI) is often used to show the blood flow inside the tumor, but CDFI is not good for some low-velocity microvessels [7]. As a novel ultrasonic technique, SMI can quickly, simply and noninvasively observe the microvascular distribution in the tumor and evaluate the microvascular perfusion [8]. Previous studies have shown that SMI can detect the blood flow signals of neovascularization in tumor and increased the sensitivity for detecting thyroid cancer. However, the results of these studies have been contradictory and the sample sizes were not enough. Therefore, the present meta-analysis aimed at evaluating the value of TI-RADS combined with SMI in distinguishing benign and malignant thyroid nodules.

## Methods

This study was conducted in accordance with the PRISMA (Preferred Reporting Items for Systematic Reviews and MetaAnalyses) guidelines, the protocol was registered in the INPLASY (INPLASY202070113), and the protocol of this meta-analysis has been published [9].

### Literature search

We searched PubMed, Web of Science, Cochrane Library, and Chinese biomedical databases from inception through February 31, 2021. The following keywords and MeSH terms were used: ["thyroid cancer" or "thyroid neoplasm" or "thyroid tumor" or "thyroid nodule "] and [“superb microvascular imaging”]. We also performed a manual search to find other potential articles.

### Selection criteria

The following 4 criteria were required for each study: (1) the study design must be a clinical cohort study or diagnostic test, (2) the study must relate to the accuracy of TI-RADS and SMI for the differential diagnosis of benign and malignant thyroid nodules, (3) all thyroid nodules were histologitally confirmed after SMI, and(4) published data in the fourfold (2×2) tables must be sufficient. If the study did not meet all of these inclusion criteria, it was excluded. The most recent publication or the publication with the largest sample size was included when the authors published several studies using the same subjects.

### Data extraction

Relevant data were systematically extracted from all included studies by two researchers using a standardized form. The researchers collected the following data: the first author’s surname, publication year, language of publication, study design, sample size, number of lesions, source of the subjects, "gold standard," and diagnostic accuracy. The true positives (TP), true negatives (TN), false positives (FP), and false negatives (FN) in the fourfold (2 x 2) tables were also collected.

### Quality assessment

Methodological quality was independently assessed by two researchers based on the quality assessment of studies of diagnostic accuracy studies (QUADAS) tool [10]. The QUADAS criteria included 14 assessment items. Each of these items was scored as "yes" (2), "no" (0), or "unclear"(1). The QUADAS score ranged from 0 to 28, and a score≧22 indicated good quality.

### Statistical analysis

The STATA version 14.0 (Stata Corp, College Station, TX, USA), Meta-Disc version 1.4 (Universidad Complutense, Madrid, Spain), and MedCalc version 15.2.2 (MedCalc Software, Ostend,Belgium) softwares were used for meta-analysis. We calculated the pooled summary statistics for sensitivity (Sen), specificity (Spe) with their 95%confidence intervals (CIs). The summary receiver operating characteristic (SROC) curve and corresponding area under the curve (AUC) were obtained. We compared the two AUC areas of single TI-RADS and TI-RADS combined with SMI. We conducted Begger’s funnel plots and Egger’s linear regression tests to investigate publication bias.

## Results

### Characteristics of included studies

**Fig 1:**
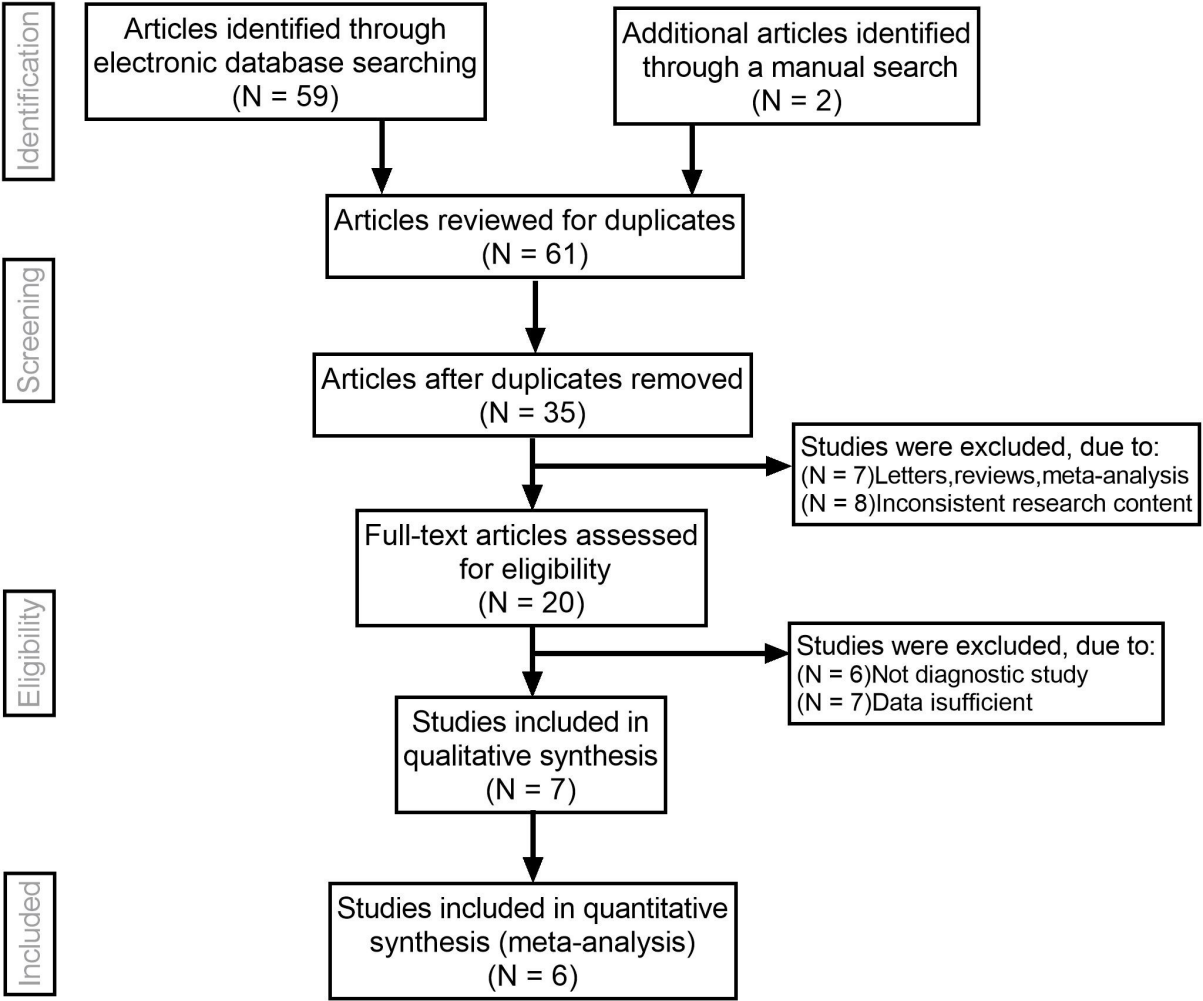
Flow chart of literature search and study selection. Six studies were included in this meta-analysis.

Initially, the searched keywords identified 61 articles. We Reviewed the titles and abstracts of all articles and excluded 40 articles; full texts and data integrity were also reviewed and 15 were further excluded. Finally, 6 studies that met all inclusion criteria were included in this meta-analysis [11–16]. Fig 1 showed the selection process of eligible articles. A total of 408 thyroid malignant nodules and 496 thyroid benign nodules were assessed. We summarized the study characteristics and methodological quality in Table 1. The QUADAS scores of all included studies were ≥22.

**Table 1:** Baseline characteristics and methodological quality of all included studies.

First author	Year	Country	Language	Sample size	Age(Years)	Instrument	TI-RADS 2×2 table					TI-RADS+SMI 2×2 table				QUADAS score
							TP	FP	FN	TN	TP		FP	FN	TN	
Zhao YF [11]	2019	China	Chinese	296	——	Toshiba Ap1io500	109	30	35	122	124		17	15	140	23
Huang JY [12]	2019	China	Chinese	68	40.1±11.1	Toshiba Ap1io500	41	6	2	19	39		3	4	22	23
Zhang PS [13]	2019	China	Chinese	121	55.6±9.0	Toshiba Ap1io500	27	6	16	72	36		4	7	74	22
LI YH [14]	2017	China	Chinese	254	39.0±16.5	Toshiba Ap1io500	64	47	9	134	65		16	18	165	23
Ahn HS [15]	2018	Korea	English	52	51.6±11.2	Toshiba Ap1io500	21	3	5	23	25		7	1	19	24
Kong J [16]	2017	China	English	113	42(20–75)	Toshiba Ap1io400	62	4	17	30	63		5	16	29	24

TP true positive, TN true negative, FP false positive, FN false negative, QUADAS the quality assessment of studies of diagnostic accuracy studies.

### Quantitative data synthesis

**Fig 2:**
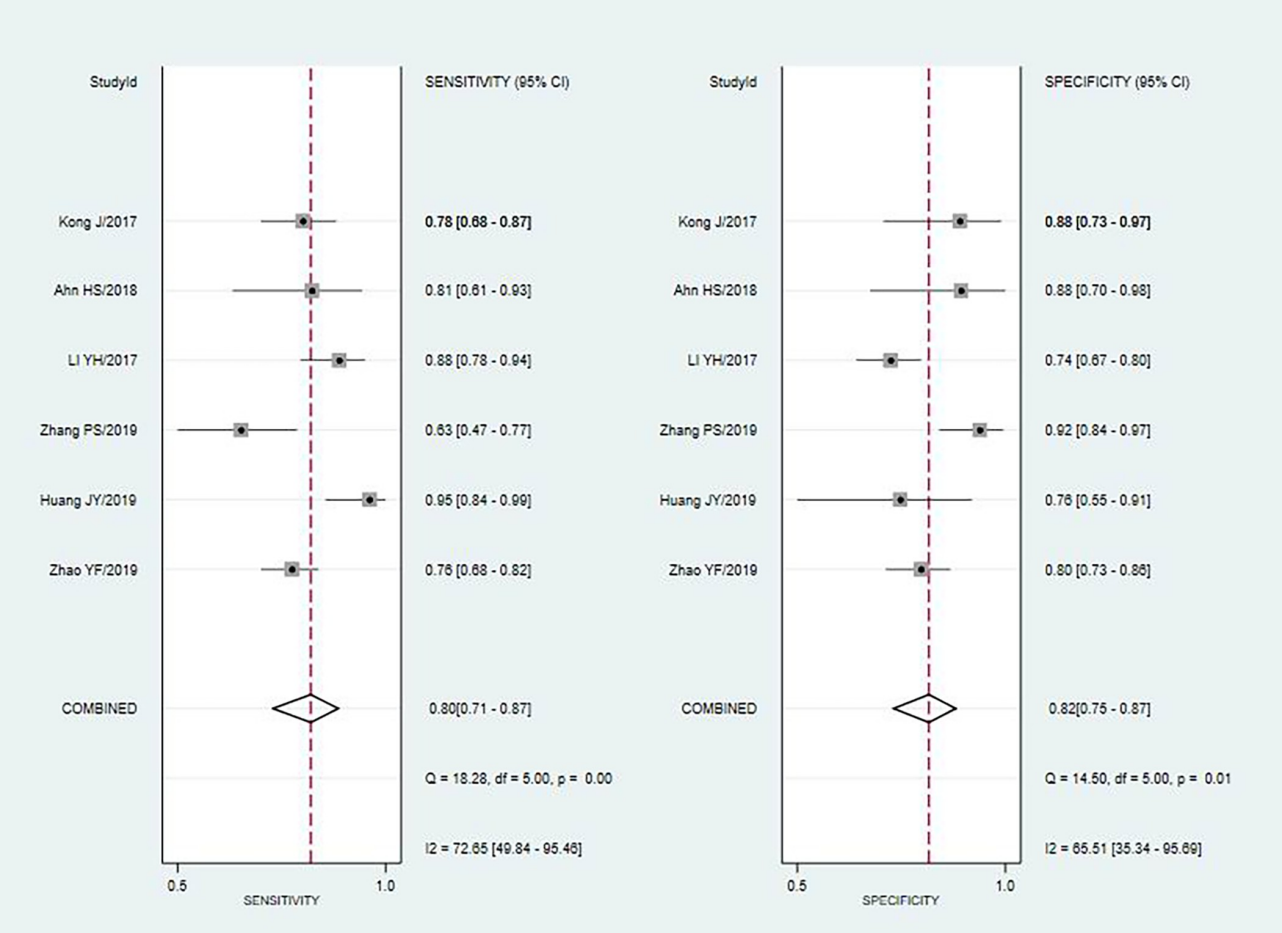
Forest plots for the accuracy of TI-RADS for the diagnosis of thyroid nodules.

**Fig 3:**
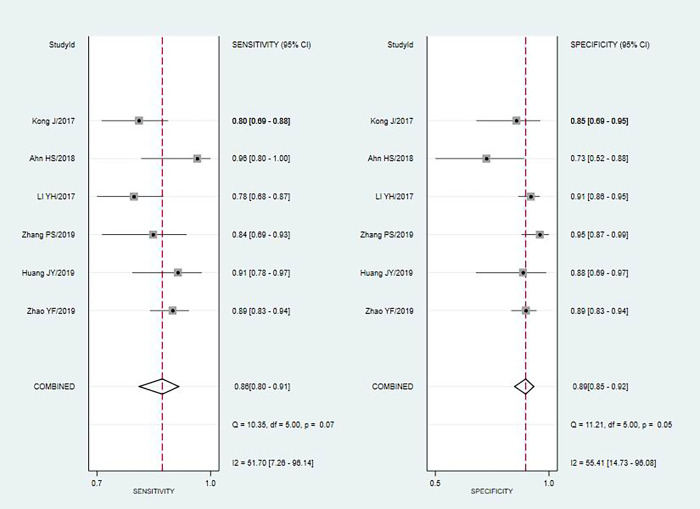
Forest plots for the accuracy of TI-RADS combined with SMI for the diagnosis of thyroid nodules.

**Fig 4:**
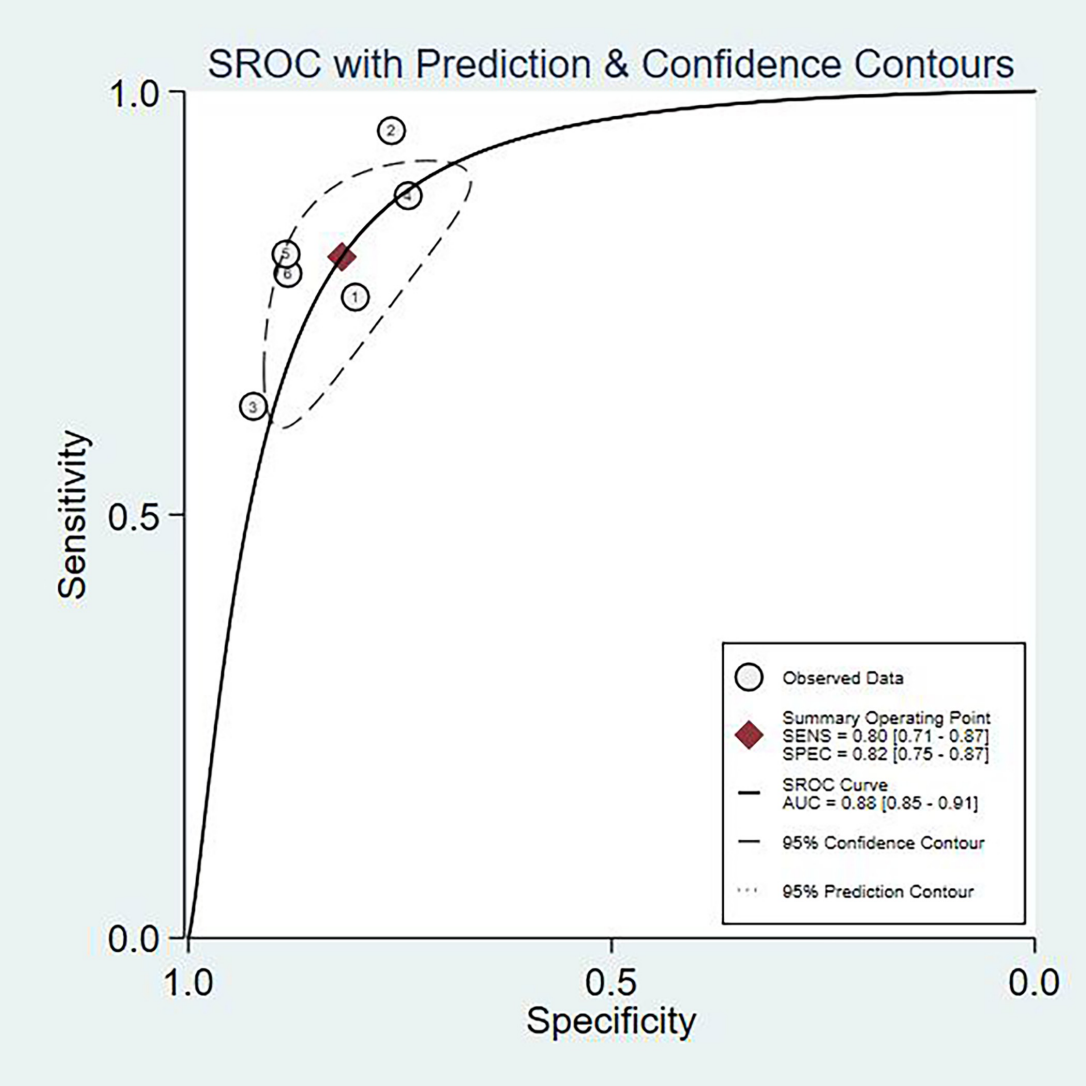
SROC curve for the accuracy of TI-RADS in the diagnosis of thyroid nodules. SROC summary receiver operator characteristic, AUC area under curve.

**Fig 5:**
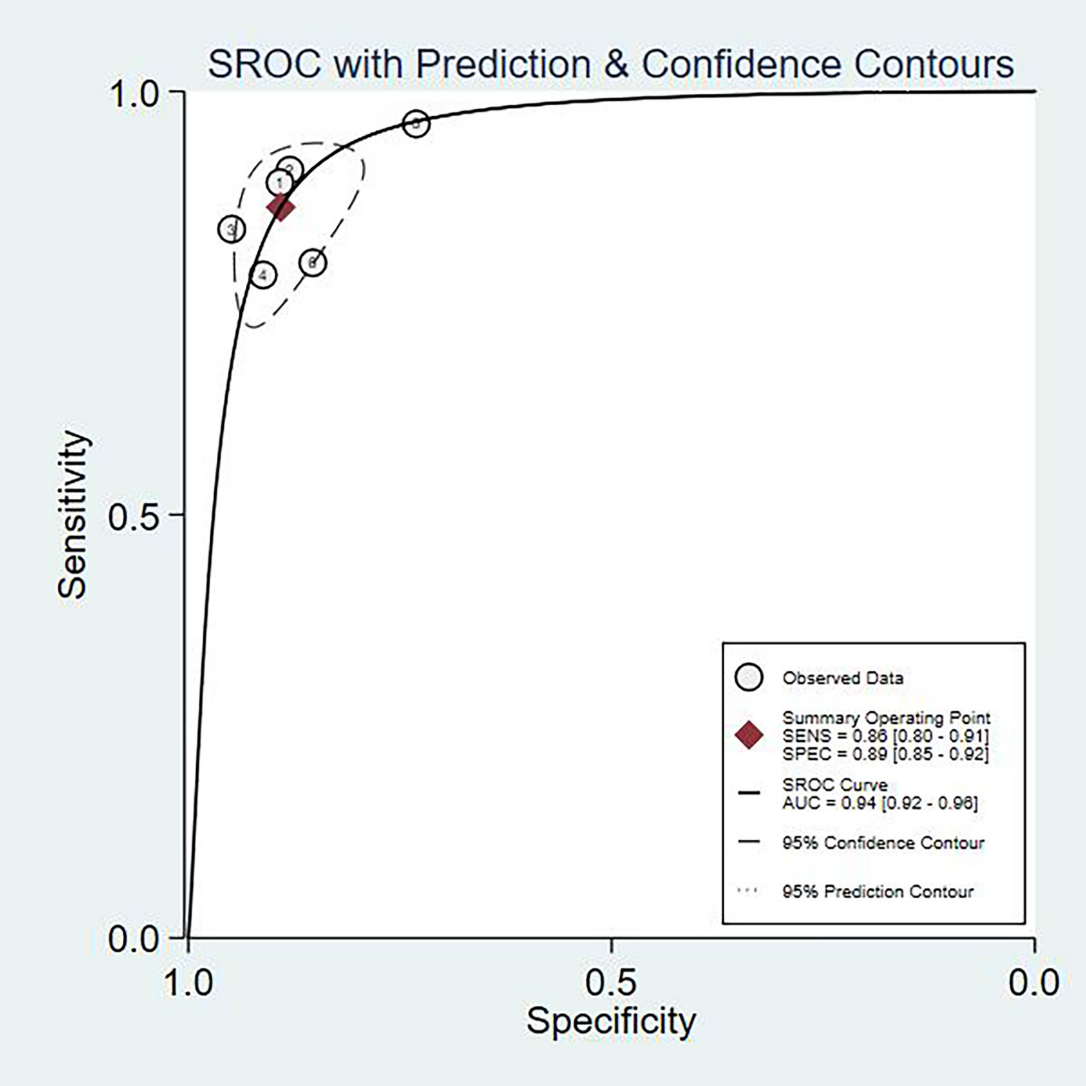
SROC curve for the accuracy of TI-RADS combined with SMI in the diagnosis of thyroid nodules. SROC summary receiver operator characteristic, AUC area under curve.

**Fig 6:**
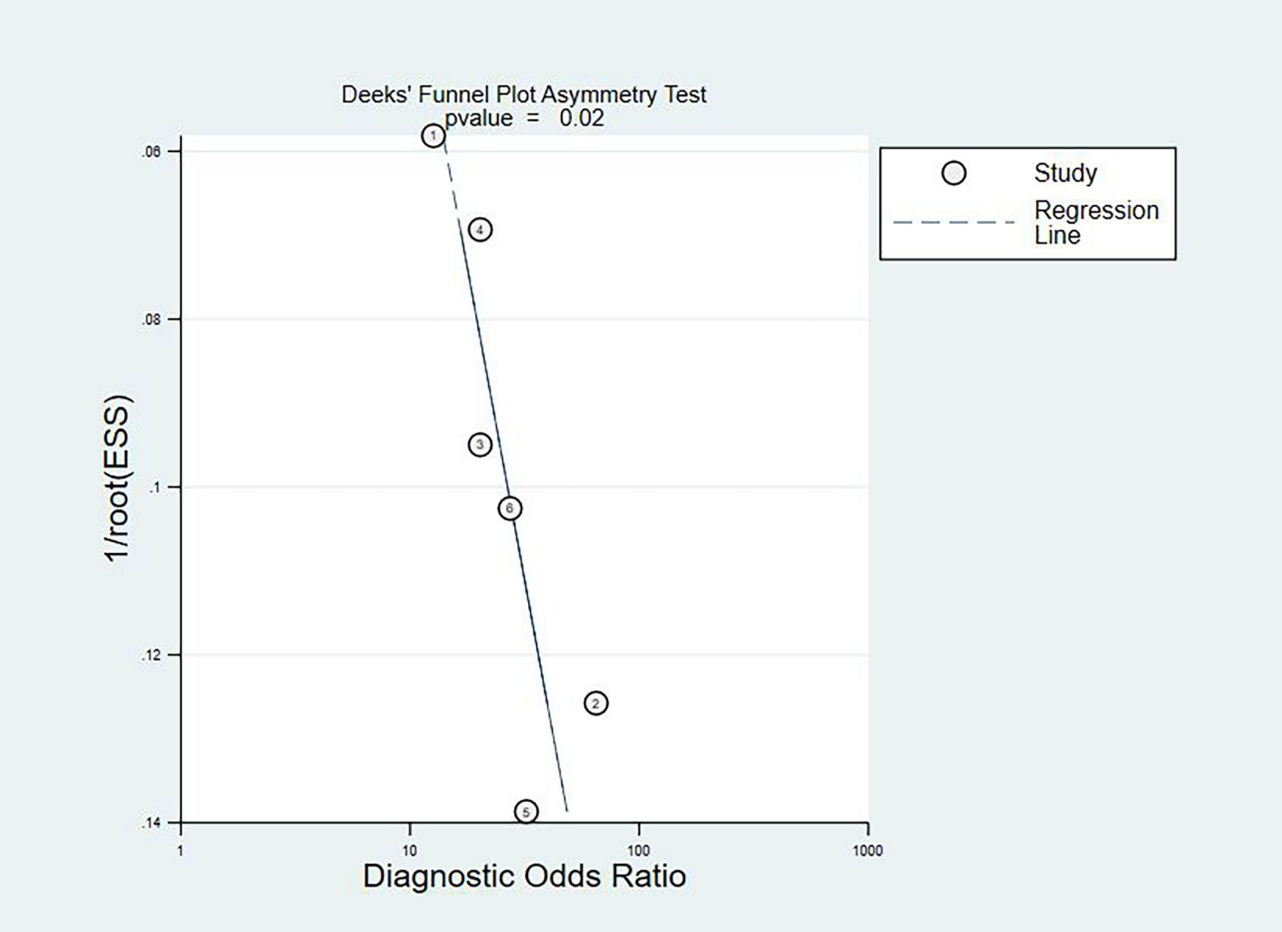
Begger’s funnel plot of publication bias on the pooled OR. No publication bias was detected in this meta-analysis.

The fixed effects model was used due to diagnostic test and not very obvious heterogeneity among the studies. The pooled Sen and Spe of TI-RADS were 0.80(95%CI = 0.71–0.87) and 0.82(95%CI = 0.75–0.87) [Fig 2]; The pooled Sen and Spe of TI-RADS combined with SMI were 0.88 (95%CI = 0.80–0.91) and 0.89 (95%CI = 0.85–0.92) [Fig 3]. The areas under the SROC curve of TI-RADS and TI-RADS combined with SMI were 0.8874 (SE = 0.0165) [Fig 4] and 0.9415 (SE = 0.0102) [Fig 5], between which there was significant difference(Z = 2.789; SE = 0.0194; p = 0.0053). We found no evidence of obvious asymmetry in the Begger’s funnel plots (Fig 6). Egger’s test also did not display strong statistical evidence for publication bias (t = 0.06, P = 0.96).

## Discussion

Thyroid nodule is a common clinical disease, and its accurate differentiation has important guiding significance for clinical decision-making. High resolution ultrasound plays an important role in the differential diagnosis of thyroid nodules [17]. Although ultrasound features of thyroid malignant nodules, such as low echo, unclear margin, microcalcification and aspect ratio > 1, increase the risk of evaluation of malignant nodules, no single ultrasound feature can independently diagnose malignant nodules [18]. The growth of tumor is accompanied by the increase of blood vessels, and the blood flow distribution patterns of thyroid benign and malignant nodules are also different [19]. Although the value of color Doppler flow pattern in the diagnosis of benign and malignant thyroid nodules remains controversial, many scholars believe that the distribution of blood vessels and blood flow characteristics in thyroid nodules have certain reference value in the differential diagnosis of benign and malignant thyroid nodules [20,21]. SMI technology is based on the high-resolution Doppler technology, using the up-market ultrasonic diagnostic equipment of Aplio series to build "high-density beamformer" and "real-time application platform", and imaging the low flow velocity blood flow with a higher frame rate. Traditional Doppler ultrasound uses filtering technology to eliminate noise and motion artifacts, resulting in the loss of low-speed blood flow information. SMI technology can identify the noise generated by blood flow and tissue movement, and use adaptive calculation method to display the real blood flow information [8].

Present studies have revealed that SMI was able to identify low-velocity blood flow without being affected by motion artifacts, and as an adjunct to grayscale US, SMI showed a significantly improved diagnostic performance in differentiating between benign and malignant thyroid nodules than either US alone or US with CDFI [16]. Nevertheless, SMI will not replace but can only compliment conventional ultrasound. Although considered as a potentially useful imaging tool, SMI is still not widely used in clinical practice, and few reports have discussed its use in the evaluation of breast tumors. In the present meta-analysis, we systematically evaluated the technical performance and accuracy of TI-RADS combined with SMI for differential diagnosis of benign and malignant thyroid nodules. The 6 independent studies were included, and a total of 408 thyroid malignant nodules and 496 thyroid benign nodules were assessed. The pooled Sen and Spe of TI-RADS were 0.80 and 0.82; The pooled Sen and Spe of TI-RADS combined with SMI were 0.88 and 0.89. The areas under the SROC curve of TI-RADS and TI-RADS combined with SMI were 0.8874 and 0.9415. The diagnostic efficiency of TI-RADS combined with SMI for distinguishing benign and malignant thyroid nodules was more better than TI-RADS alone. In other words, SMI may be a good tool to diagnose thyroid nodules.

Despite the demonstrated diagnostic accuracy of TI-RADS combined with SMI for thyroid nodule, our study has certain limitations. First, owing to the relatively small sample sizes and low level of quality of the included studies, there were insufficient data to assess the accuracy of SMI. Moreover, the retrospective nature of a meta-analysis can lead to subject selection bias. Importantly, the majority of included studies originated from China, which may adversely affect the reliability and validity of our results.

In conclusion, our meta-analysis suggests that TI-RADS combined with SMI may have high diagnostic accuracy in distinguishing benign and malignant thyroid nodules, and SMI may be a good tool to diagnose thyroid nodule. However, due to the limitations, further detailed studies are required to confirm the present findings.

## Supporting information

S1 Checklist PRISMA 2009 checklist. (DOC)
