# Supplementary material for: The value of TI-RADS combined with superb micro-vascular imagine in distinguishing benign and malignant thyroid nodules: A meta-analysis
Source: PLoS One. 2022 Jan 18;17(1):e0261521. doi: 10.1371/journal.pone.0261521 (PMC8765637; doi:10.1371/journal.pone.0261521)
Supplement: S1 Checklist — (DOC) [file pone.0261521.s001.doc]

| **Section/topic** | **#** | **Checklist item** | **Reported on page #** |
| --- | --- | --- | --- |
| **TITLE** | | |  |
| Title | 1 | The value of TI-RADS combined with superb micro-vascular imagine in distinguishing benign and malignant thyroid nodules：a meta-analysis | 1 |
| **ABSTRACT** | | |  |
| Structured summary | 2 | This meta-analysis aimed to evaluate the value of thyroid imaging report and data system (TI-RADS) combined with superb micro-vascular imagine technique(SMI) in distinguishing benign and malignant thyroid nodules. We searched PubMed, Web of Science, Cochrane Library, and Chinese biomedical databases from inception through February 31, 2021. Meta-analysis was conducted using STATA version 14.0 and Meta-Disc version 1.4 softwares. We calculated the summary statistics for sensitivity(Sen), specificity(Spe), and receiver operating characteristic (SROC) curve. Six studies that met all inclusion criteria were included in this meta-analysis. A total of 408 thyroid malignant nodules and 496 thyroid benign nodules were assessed. All thyroid nodules were histologically confirmed after SMI. The pooled Sen and Spe of TI-RADS were 0.80(95%CI=0.71-0.87) and 0.82(95%CI=0.75-0.87); The pooled Sen and Spe of TI-RADS combined with SMI were 0.88 (95%CI=0.80-0.91) and 0.89 (95%CI=0.85-0.92). The areas under the SROC curve of TI-RADS and TI-RADS combined with SMI were 0.8874(SE=0.0165) and 0.9415(SE=0.0102), between which there was significant difference(Z=2.789; SE=0.0194; p=0.0053). Our meta-analysis indicates that TI-RADS combined with SMI may have high diagnostic accuracy, and is more effective than single TI-RADS in distinguishing benign and malignant thyroid nodules. | 2 |
| **INTRODUCTION** | | |  |
| Rationale | 3 | As a novel ultrasonic technique, SMI can quickly, simply and noninvasively observe the microvascular distribution in the tumor and evaluate the microvascular perfusion[8]. Previous studies have shown that SMI can detect the blood flow signals of neovascularization in tumor and increased the sensitivity for detecting thyroid cancer. However, the results of these studies have been contradictory and the sample sizes were not enough. Therefore, the present meta-analysis aimed at evaluating the value of TI-RADS combined with SMI in distinguishing benign and malignant thyroid nodules. | 2 |
| Objectives | 4 | Therefore, the present meta-analysis aimed at evaluating the value of TI-RADS combined with SMI in distinguishing benign and malignant thyroid nodules. | 2 |
| **METHODS** | | |  |
| Protocol and registration | 5 | This study was conducted in accordance with the PRISMA (Preferred Reporting Items for Systematic Reviews and MetaAnalyses) guidelines, the protocol was registered in the INPLASY (INPLASY202070113), and the protocol of this meta-analysis has been published. | 4 |
| Eligibility criteria | 6 | The following 4 criteria were required for each study: (1) the study design must be a clinical cohort study or diagnostic test, (2) the study must relate to the accuracy of TI-RADS and SMI for the differential diagnosis of benign and malignant thyroid nodules, (3) all thyroid nodules were histologitally confirmed after SMI, and(4) published data in the fourfold (2×2) tables must be sufficient. If the study did not meet all of these inclusion criteria, it was excluded. The most recent publication or the publication with the largest sample size was included when the authors published several studies using the same subjects. | 3 |
| Information sources | 7 | We searched PubMed, Web of Science, Cochrane Library, and Chinese biomedical databases from inception through February 31, 2021. | 3 |
| Search | 8 | The following keywords and MeSH terms were used: ["thyroid cancer" or "thyroid neoplasm" or "thyroid tumor" or "thyroid nodule "] and [“superb microvascular imaging”]. We also performed a manual search to find other potential articles. | 3 |
| Study selection | 9 | Initially, the searched keywords identified 61 articles. We Reviewed the titles and abstracts of all articles and excluded 40 articles; full texts and data integrity were also reviewed and 15 were further excluded. Finally, 6 studies that met all inclusion criteria were included in this meta-analysis | 4 |
| Data collection process | 10 | Relevant data were systematically extracted from all included studies by two researchers using a standardized form. | 4 |
| Data items | 11 | The researchers collected the following data: the first author's surname, publication year, language of publication, study design, sample size, number of lesions, source of the subjects, "gold standard," and diagnostic accuracy. The true positives (TP), true negatives (TN), false positives (FP), and false negatives (FN) in the fourfold (2 x 2) tables were also collected. | 3 |
| Risk of bias in individual studies | 12 | Methodological quality was independently assessed by two researchers according to a tool for the quality assessment of methodological index for non-randomized studies(MINORS) . | 3 |
| Summary measures | 13 | Methodological quality was independently assessed by two researchers based on the quality assessment of studies of diagnostic accuracy studies (QUADAS) tool[10]. The QUADAS criteria included 14 assessment items. Each of these items was scored as "yes" (2), "no" (0), or "unclear"(1). The QUADAS score ranged from 0 to 28, and a score≧22 indicated good quality. | 4 |
| Synthesis of results | 14 | The STATA version 14.0 (Stata Corp, College Station, TX, USA), Meta-Disc version 1.4 (Universidad Complutense, Madrid, Spain), and MedCalc version 15.2.2 (MedCalc Software, Ostend,Belgium) softwares were used for meta-analysis. We calculated the pooled summary statistics for sensitivity (Sen), specificity (Spe) with their 95%confidence intervals (CIs). The summary receiver operating characteristic (SROC) curve and corresponding area under the curve (AUC) were obtained. We compared the two AUC areas of single TI-RADS and TI-RADS combined with SMI. | 3 |

Page 1 of 2

| **Section/topic** | **#** | **Checklist item** | **Reported on page #** |
| --- | --- | --- | --- |
| Risk of bias across studies | 15 | We conducted Begger’s funnel plots and Egger’s linear regression test to investigate publication bias. | 4 |
| Additional analyses | 16 | In order to evaluate the influence of single study on the overall estimate, sensitivity analysis was performed. | 4 |
| **RESULTS** | | |  |
| Study selection | 17 | 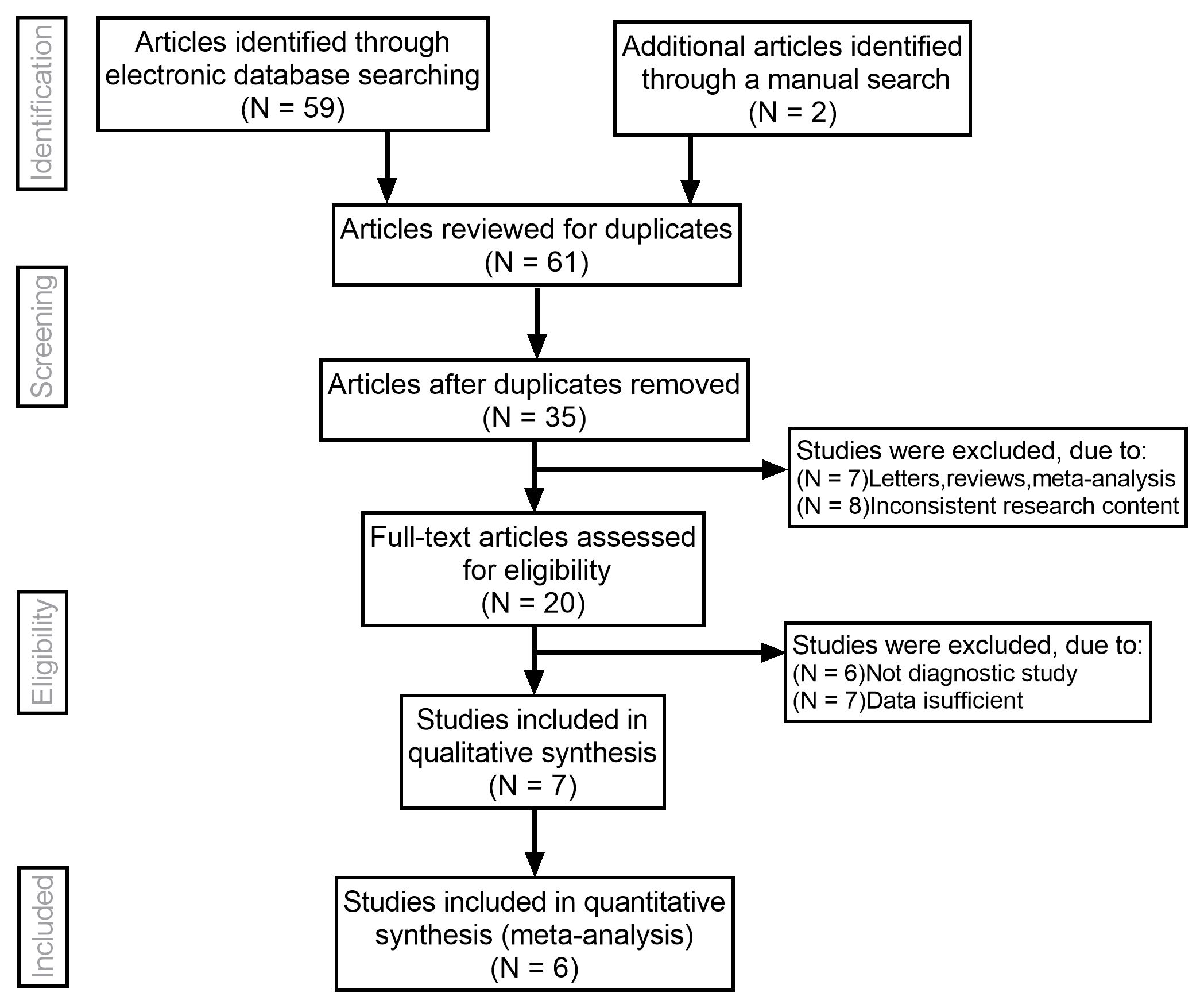 | 4 |
| Study characteristics | 18 | A total of 408 thyroid malignant nodules and 496 thyroid benign nodules were assessed. We summarized the study characteristics and methodological quality in Table1. | 4 |
| Risk of bias within studies | 19 | The QUADAS scores of all included studies were ≥22. | 4 |
| Results of individual studies | 20 | 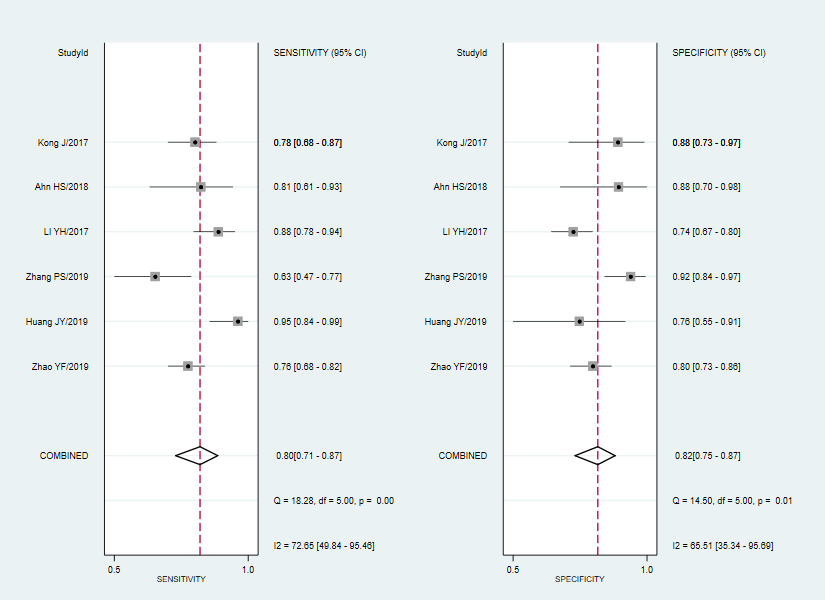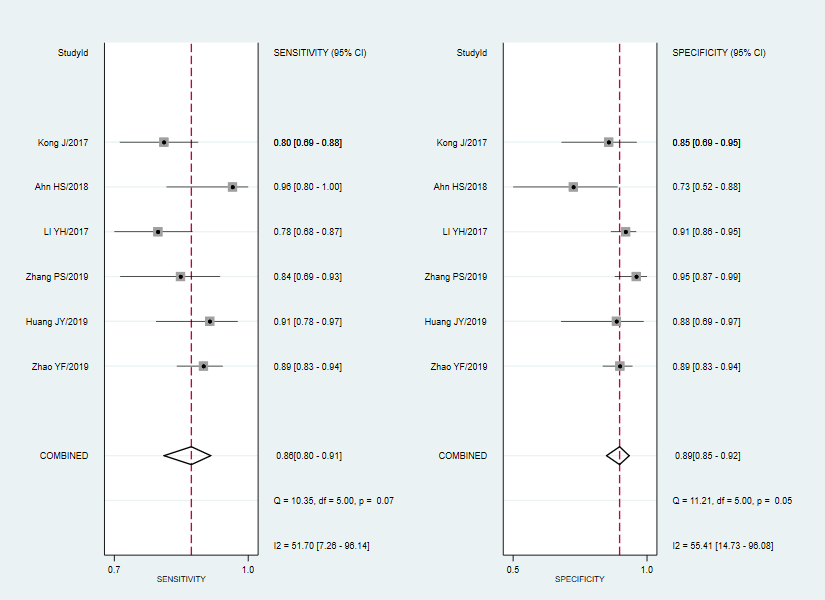 | 4 |
| Synthesis of results | 21 | The pooled Sen and Spe of TI-RADS were 0.80(95%CI=0.71-0.87) and 0.82(95%CI=0.75-0.87)[Figure 2]; The pooled Sen and Spe of TI-RADS combined with SMI were 0.88 (95%CI=0.80-0.91) and 0.89 (95%CI=0.85-0.92)[Figure 3]. The areas under the SROC curve of TI-RADS and TI-RADS combined with SMI were 0.8874 (SE=0.0165)[Figure 4] and 0.9415 (SE=0.0102)[Figure 5], between which there was significant difference(Z=2.789; SE=0.0194; p=0.0053). | 4 |
| Risk of bias across studies | 22 | Egger’s test also did not display strong statistical evidence for publication bias. | 4 |
| Additional analysis | 23 | Sensitivity analysis was carried out, and none of them caused obvious interference to the results. | 4 |
| **DISCUSSION** | | |  |
| Summary of evidence | 24 | In conclusion, our meta-analysis suggests that TI-RADS combined with SMI may have high diagnostic accuracy in distinguishing benign and malignant thyroid nodules, and SMI may be a good tool to diagnose thyroid nodule. | 6 |
| Limitations | 25 | First, owing to the relatively small sample sizes and low level of quality of the included studies, there were insufficient data to assess the accuracy of SMI. Moreover, the retrospective nature of a meta-analysis can lead to subject selection bias. Importantly, the majority of included studies originated from China, which may adversely affect the reliability and validity of our results. | 6 |
| Conclusions | 26 | SMI may be a good tool to diagnose thyroid nodule. | 6 |
| **FUNDING** | | |  |
| Funding | 27 | This study is supported by Liaoning Natural Science Foundation Project (20170540256). | 1 |

*From:*  Moher D, Liberati A, Tetzlaff J, Altman DG, The PRISMA Group (2009). Preferred Reporting Items for Systematic Reviews and Meta-Analyses: The PRISMA Statement. PLoS Med 6(7): e1000097. doi:10.1371/journal.pmed1000097

For more information, visit: **www.prisma-statement.org**.

Page 2 of 2
